# EBM in primary care: a qualitative multicenter study in Spain

**DOI:** 10.1186/1471-2296-12-84

**Published:** 2011-08-09

**Authors:** Carlos Calderón, Iván Sola, Rafael Rotaeche, Mèrce Marzo-Castillejo, Arturo Louro-González, Ricard Carrillo, Ana-Isabel González, Pablo Alonso-Coello

**Affiliations:** 1 Centro de Salud de Alza, Donostia-San Sebastián, Spain; 2 Iberoamerican Cochrane Center, Hospital Sant Pau, Sant Antoni Maria Claret 171, Barcelona, Spain; 3 Centro de Salud de Alza, Donostia-San Sebastián, Spain; 4 Àmbit d'Atenció Primària Costa de Ponent, Barcelona, Spain; 5 Servicio de Atención Primaria de Cambre, Cambre, Coruña, Spain; 6 EAP La Florida Sud, L'Hospitalet de Llobregat, Barcelona, Spain; 7 Centro de Salud Vicente Muzas, Área 4, Madrid, Spain; 8 CIBER de Epidemiología y Salud Pública (CIBERESP), Spain

## Abstract

**Background:**

Evidence based medicine (EBM) has made a substantial impact on primary care in Spain over the last few years. However, little research has been done into family physicians (FPs)' attitudes related to EBM. The present study investigates FPs' perceptions of EBM in the primary care context.

**Methods:**

This study used qualitative methodology. Information was obtained from 8 focus groups composed of 67 FPs from 47 health centers in 4 autonomous regions in Spain. Intentional sampling considered participants' previous education in EBM, and their experience as tutors in family medicine or working groups' members of the Spanish Society of Family Practice. Sociological discourse analysis was used with the support of the MAXqda software. Results were validated by means of triangulation among researchers and contrast with participants.

**Results:**

Findings were grouped into three main areas: 1) The tug-of-war between the "science" of EBM and "experience" in the search for good clinical practice in primary care; 2) The development of EBM sensemaking as a reaction to contextual factors and interests; 3) The paradox of doubt and trust in the new EBM experts.

The meaning of EBM was dynamically constructed within the primary care context. FPs did not consider good clinical practice was limited to the vision of science that EBM represents. Its use appeared to be conditioned by several factors that transcended the common concept of barriers. Along with concerns about its objectivity, participants showed a tendency to see EBM as the use of simplified guidelines developed by EBM experts.

**Conclusions:**

The identification of science with EBM and its recognition as a useful but insufficient tool for the good clinical practice requires rethinking new meanings of evidence within the primary care reality. Beyond the barriers related to accessing and putting into practice the EBM, its reactive use can determine FPs' questions and EBM development in a direction not always centred on patients' needs. The questioning of experts' authority as a pillar of EBM could be challenged by the emergence of new kinds of EBM texts and experts to believe in.

## Background

There is now no doubt that evidence-based medicine (EBM) plays an important role in health care. EBM was first presented in the early 1990s as a new movement orientated towards "integrating individual clinical expertise with the best available external clinical evidence from systematic research"[1]. On one hand it was about questioning non-rigorous clinical observations, physio-pathological rationale and experts' authority as reliable pillars of medical knowledge [2], and on the other, it proposed the need for new skills: formulation of well structured questions, search and retrieval of the best available evidence, and critical assessment of available medical literature. The instruments have since evolved tremendously in relation to access to and interpretation of information, and the production of evidence-based clinical practice guidelines (CPG).

Nevertheless, discussion and debate abound. From a theoretical point of view, EBM has been questioned as being reductionist and rigid with its hierarchical approach to the concept of evidence [3-5]. At a practical level, EBM has not been widely incorporated into clinical decisions, drawing attention to the potential barriers related to its implementation [6,7].

All these aspects are especially relevant in primary care (PC). Studies undertaken in several countries have shown that family physicians (FP) generally share a positive attitude towards EBM but they also emphasize the importance of certain obstacles to its implementation. The most important of these barriers appears to be the lack of time, training and resources available [8-11] and the complexity and individuality of clinical practice [12,13]. EBM training has increased in Spain over the last decade, and the new teaching curriculum in family medicine promotes awareness of EBM and the use of Internet resources in clinical practice. However, only a few local studies have studied FPs' thoughts about EBM [14]. We therefore considered it relevant to carry out a two-phase project, described in a previous publication [15]. Here we present the results of a qualitative multicenter study that aimed to investigate in depth FPs' attitudes and perceptions to EBM in our setting.

## Methods

Qualitative methods have proved specially appropriate in the approach to perceptions, beliefs and attitudes in studies related to EBM in PC [14,16-20]. In this qualitative study focused on health services research [21,22] we used group interviews [23,24] to generate information. We selected a total of 8 focus groups with 67 FPs working in 47 different public health centres of 4 autonomous regions in Spain (Galicia, Euskadi, Madrid and Catalonia) (Table 1). Two of the groups included working group members of the Spanish Society of Family Practice (GS1, GS2) and two included family medicine tutors (GT1, GT2). Two of the four remaining groups had some type of EBM training (GTR1, GTR2) and two did not (GTR3, GTR4).

**Table 1:** Participants

Groups	N° participants	N° involved Health Centres	Gender		Age		Years of work	
			**men**	**women**	**mean**	**range**	**mean**	**range**

**semFYC groups** **Madrid (GS1)**	9	7	6	3	40	30-49	17	16-24

**semFYC groups** **Barcelona (GS2)**	6	6	4	2	46	38-55	17	7-30

**Tutors** **Donostia (GT1)**	7	7	2	5	47	45-54	18	8-21

**Tutors** **Barcelona (GT2)**	12	3	8	4	44	35-51	18	7-28

**FP with training** **Barcelona (GTR1)**	12	7	5	7	35	28-43	8	2-18

**FP with training** **Donostia (GTR2)**	9	7	5	4	49	35-58	21	7-30

**FP without training** **Coruña (GTR3)**	7	6	4	3	46	30-53	24	18-32

**FP without training** **Madrid (GTR4)**	5	4	3	2	35	32-41	7	3-10

**Total**	67	47	37	30	39	28-58	16	2-32

Participants - 37 men (M) and 30 women (W) - were intentionally selected and the recruiting process stressed that the study was not an attempt to evaluate their knowledge about EBM. Its independent nature was also made clear. Confidentiality was guaranteed and a statement of informed consent was obtained from all participants. The Ethics Committee at Hospital de la Santa Creu i Sant Pau in Barcelona approved the study.

The focus groups met between October 2007 and July 2008 in health care centres and health care institutions. Meetings lasted between one and two hours and a moderator and an observer were present. The questionnaire was semi-structured, and it was modified and adapted according to the findings and group dynamics [23]. All sessions were recorded. The two researchers made notes in a field diary after each meeting.

Information generated was recorded in digital format. Material was transcribed verbatim. It was verified by the interviewers and field notes were added. Analysis was done following the model of sociological discourse analysis [25,26]. Using this model, the reading, organization and interpretation of data does not come from a fragmentation of texts but from a more global view, keeping in mind the context in which data were generated. The findings were identified in accordance with the study objectives and mapped firstly for each of the groups and also contrasted with the others taking into account their composition characteristics. Finally some common areas and axes were built to synthesize the main results. Each step in the process of interpretation and synthesis required iterative revision of texts to identify and code the main patterns and categories, and to corroborate the findings. The MAXqda program was used to organize data and to select the most representative verbatim statements. The analytical process and results were discussed and consensus was reached by triangulation among researchers, and the preliminary report was also sent to -and accepted by- the participants ("member checking"), as procedures to improve the validity of the study [27].

## Results

Analysis of the information collected showed that there were not clear patterns linked to the characteristics of the groups. Perhaps due to the variability in training and knowledge about EBM within some of them, or because the arisen concerns and narratives were not necessarily related to this factor, the main findings emerged across the different groups and could be classified into three areas. As these areas were inter-related, an overall interpretation should be kept in mind.

### 1) EBM, science, experience and good clinical practice in PC

**Figure 1:**
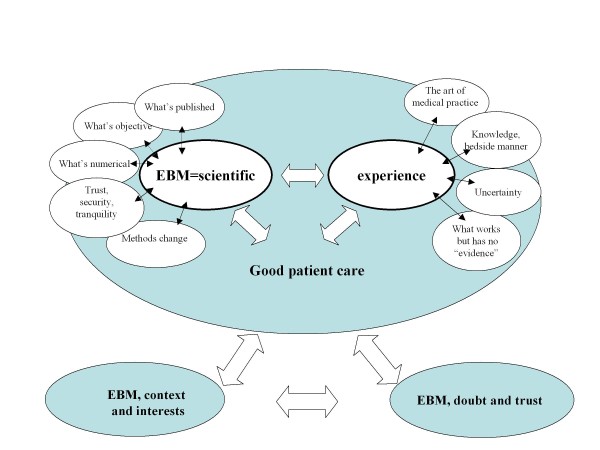
**EBM: science, experience and good patient care**.

We found that the current status and importance of EBM was widely recognized but rarely concretely defined. EBM tended to be identified through certain features related to *"science" *although this identification was often applied "by defect", that is, through components of professional practice that are "outside" the scope of EBM and grouped in the category "*experience"*. In both cases arguments and questions sought justification in *"good patient care" *(Figure 1).

Identifying EBM with "science" was mainly linked to the requirements for *objectivity *when reviewing *published *medical literature. In this sense, EBM generated *security, confidence and tranquillity*. EBM as "science" was occasionally linked to the accuracy of numbers, as opposed to the imprecision of most of the symptoms expressed by patients in the PC context.

*"Well, it's similar.... I'm thinking of scientific articles, or scientific publications that allow you to improve a bit daily practice with knowledge that is based on evidence." (M1)(GTR1)*.

*"It also at times gives me a sense of security. When they say something is so, I believe it is so. The problem is that I believe there is still much not known..."(W3) (GT1)*.

"I believe it's the future, and actually as there is more and more information on the Internet EBM will be more powerful. There's not going to be any other way to relate. Because it's giving you results with numbers, and that's what we doctors want" (M1)(GS1)

Also from the perspective of "science", EBM was seen in some discourses as a *method *of reviewing one's habitual clinical work in a critical manner and posing questions to be answered based on findings from published studies.

*"I insist on...that it is an instrument, and agree in the sense that it is an instrument that is useful especially as a method, as W4 was saying, to ask structured questions and try to find the answers...." (M2)(GS2)*.

The role of EBM in clinical practice was, however, repeatedly seen as relative and even questionable. Knowledge accumulated over years of treating patients in uncertain situations was mentioned as an important component of professional work, confronting certain perceptions of EBM which were seen as dogma. *"Experience" *therefore came up in all the groups in contraposition/complement to EBM.

*"-...The matter is that... I know that EBM is important, but you also have to place before it the experience you have, don't you? (W2)*.

*- You can't work based only on the evidence! First come your experience and knowledge, until you are proved wrong, doesn't it?(W4)"(GT1)*.

*"- In addition there is the experience. It's probably part of the art....It's our work. In PC, our work is the clinical interview. We have to know, aside from diagnosing the patient, what he or she feels, and empathize with the person.. (M1).-...One sees it at times as dogma... what I mean is that not all patients are the same and many times you are in front of a patient that EBM says has this or that, and you don't see it! Experience is also worth a lot!" (W4)(GTR2)*.

From this perspective, "experience" was related to the "art" of medicine, to the management of uncertainty, and to empathy in communication with the patient. All these aspects were considered especially important in PC and different from other specialties located at Hospitals. The need to respond to clinical practice problems that have no clear answers in the CPGs stressed the importance of the meaning of "experience" as a necessary component of the "*good patient care*".

"-In spite of clinical evidence being there and telling you what to do, then you clash with the subjectivity of the relationship with the patient...and that also produces a certain frustration and leads you to think that EBM does not work .... (M3)

*- Anyway I believe that this same forum with oncologists would be different, because in other branches of medicine they live in the most absolute certainty...(M5)"(GS1)*.

"- I think that medicine is not only a science. It's an art. Since it's also an art, it is clear that not everything can be based on scientific evidence... and that many times you need to negotiate, make pacts, there's a lot of that. Medicine does not have to be 2 × 2 = 4. Medicine is something else, isn't it?... (W2)

*- But then there are many small consultations for which we don't have guidelines! There are no guidelines for all consultations. There may be some sort of orientation, but everything is not in the guidelines written according to EBM.(W7)" (GT2)*.

### 2) EBM: Context and interests

**Figure 2:**
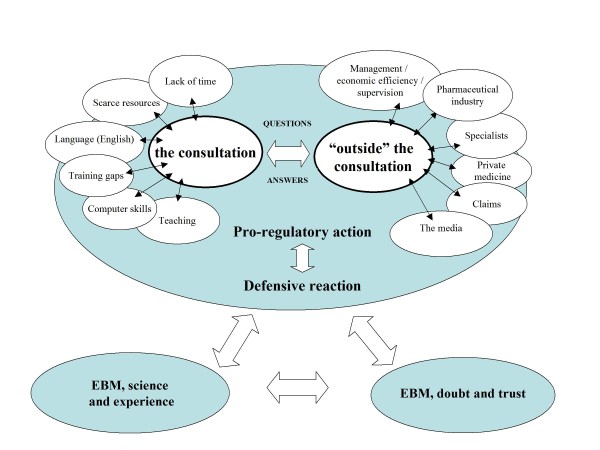
**EBM: context and interests**.

Perceptions about the context in which physicians work made up a second area of results that included two schematic sub-areas (Figure 2). The first of these represented patient care within the *consultation room*, where clinical questions arise and decisions are made. The second, necessarily related to the first, tried to show the diverse paths from "*outside" the consultation room*, that is, beyond the relationship with the patient, that especially condition the use of EBM in PC.

With respect to the work within the *consultation room *all groups agreed about the excessive workload and the lack of time for routine use of EBM. These obstacles were added to other difficulties such as the lack of *resources, educational gaps *and limitations in areas such as *English *or *information technologies*. Under such circumstances, EBM seemed to be more widely used in the area of training.

*"... ok, it's important, but it takes up a little space in our everyday work, and above all due to lack of time, doesn't it? If you have 30-40-50 patient visits, logically you're not going to do anything about EBM at that time. " (M2)(GTR1)*.

*"...for me the language is also important; if they give you a guideline in Spanish, or in English, it makes a difference..." (W1)(GTR4)*.

*"We really do what we can in the daily office visits, but when the Residents are there I think we make an effort to really apply EBM, in order to train them." (W4)(GT2)*.

From "*outside" the consultation *one of the conditional factors for using the EBM and CPGs was their linkage to economic efficiency and control promoted by management.

"It seems that EBM has been focused a little on the drugs we give or we don't give, because behind that there is a cost, and we also have the manager that harps on us about it..."(W5)(GTR1)

"...Because you feel they are controlling your work! They compare what you do with respect to the guidelines or the evidence. I think there is a sensation of threat, or even of resistance to... I've always done it this way (M2)." (GTR2)

References to pressure from the *pharmaceutical industry *constituted the "external factor" that most repeatedly appeared in connection with the use of EBM. EBM seemed to represent an important tool to neutralize its influence, helping to reveal the biases and gaps in misleading advertising.

*"...because of course, the truth is that pharmaceutical companies or large institutions blatantly fool us. ...So EBM has his base on something that has been demonstrated before and that doesn't camouflage in statistics." (M2)(GT1)*.

*"I believe industry does a good job. They are private institutions whose goal is to make money. Our job is to be able to filter that a bit. To begin with, we must not believe a word of what the industry says." (M1)(GS2)*.

EBM was also perceived as a useful instrument to curb the influence of *specialized healthcare *and the *private sector*, allowing the physicians to question their criteria. And finally, the influence of the *media *and possible claims from the *patients *were also "external" motives to accept the security that EBM provides in case of unjustified requests.

"- The problem is also that for example, we have to prescribe things referred from specialists... EBM helps in this, and what I do is, based on the evidence, say to my patient that there is no scientific evidence that has proven this, and sometimes I do this so as not to continue prescribing a medication.(W6)

*-...This has brought to my mind the matter of the risk that we often have of making medical mistakes, don't we?... And that EBM could help you a bit, so you can feel that if you have a claim from a patient because of a medical error, at least you have based your actions on EBM! (W2)(GT2)*.

*"Yes we use it, but especially to deny treatments. To justify a decision when there is concern about it, especially in our case the medications prescribed by private physicians, don't we?" (W3)(GTR1)*.

Consequently, on one hand, EBM was seen as a "*pro-active*" instrument that helps to achieve a good practice despite clear limitations derived from the conditions of PC consultations. On the other hand, however, and often more importantly, EBM was considered a "*reactive*" resource related to interests and pressures from "*outside" the consultation*, and even from "outside" the real patients' needs.

### 3) EBM: doubt and trust

**Figure 3:**
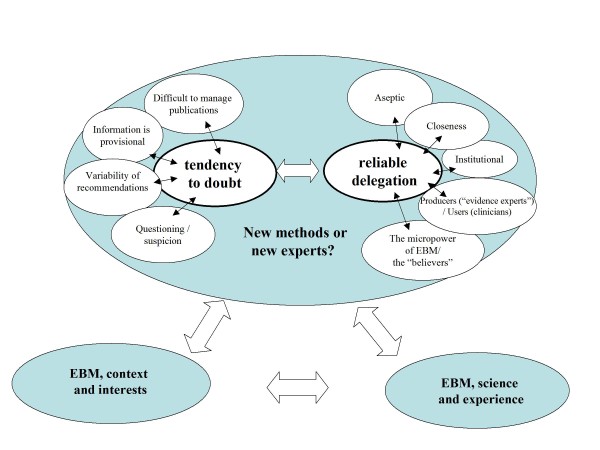
**EBM, doubt and trust**.

Findings in the discourses in the third area showed a somewhat paradoxical scenario, especially in groups that had a greater prior knowledge of EBM. As mentioned previously, perceptions of EBM included a *questioning attitude *component that led FPs to review the scientific basis of many clinical norms. This "*tendency to doubt*" (Figure 3) was heightened by the unlimited growth of the *published literature *which is very difficult to access and select. For this reason published evidence was often seen as *biased *or *provisional*.

*"- At times the evidence can be easily manipulated, can't it? It's not the same thing to say what is the evidence now than in 10 years time (W2)*.

- I don't dare to talk about some things... medications no!, use this!, and in a few years there's been another study and I don't know what... and then you treat the condition in a different manner (W5)." (GT1)

*"I believe this has turned us into "doubt experts", because I had not thought that one should continuously doubt." (W1)(GT2)*.

Family practitioners' doubts increased when different CPGs gave different recommendations for the same problem even though they all claimed to be based on EBM. In such situations FPs stated they looked for *reliable references *to help reduce their uncertainty. They did not want to be involved in searching and discriminating the evidence, but they needed to trust those who write GPCs and other EBM products. The grounds for trust, according to the FPs, were that guidelines should be "*aseptic" *(i.e. free of conflicts of interests related to the pharmaceutical industry, for example), have *institutional *validation, and be developed by a *close *and *well known *source.

*"I am reviewing now for a manual and there are two American guidelines that are totally contradictory. They both claim to be based on evidence and they both give type A recommendations. Which one is correct? What evidence can you trust? (M1)(GS2)*.

*"...I'm not going to check out all the studies. I have to trust someone who does that work. It makes things easier for me." (W1)(GTR3)*.

"- Who validates this? It should be the most aseptic, shouldn't it? So you imagine a very aseptic scientific association because it has nothing to do with the industry or with the administration, although this is not always so, but well....(W3)

*- I think you also accept what comes closest. You can check some clinical guidelines, maybe you rely more on the Catalonian one because you are from Catalonia... (W1)" (GTR1)*.

Discourses from the different groups thus reflected at least two types of professionals: those that *"use EBM"*, the majority, and those that *"make EBM"*. Those that "make" EBM were considered a new kind of experts or "evidence experts", expected to produce reliable tools that are easy to apply in daily work. And among the users a new kind of "believers" also appeared, conscious of their different knowledge status.

*"I leave this in the hands of the evidence experts. That you have to recommend aspirin here and recommend it there, I believe him! I don't wonder how he/she reached that conclusion nor if there is evidence. I believe it." (M1)(GTR2)*.

*"I don't pretend to be an expert. I think it's ok that people request guides that have been thoroughly gone over. At least let me know if it has an A1 level or a B2 level, don't tell me where it comes from. Most of us are believers, what the (EBM) group tells me I believe it...". (M5)(GS1)*.

The *doubt *and *trust *dimensions revealed a paradox based on diverse arguments. FPs tended to be suspicious of the vast amount and disparity of material published under the EBM label, and thus somewhat sceptical to change. However, their behaviour also showed an increasing demand for EBM in an "easy", "clear", "cookbook type" CPG format. Their trust appeared to depend more on the degree of closeness with the new "experts" involved in their development of EMB than on the use of a different method for the acquisition and application of knowledge.

## Discussion

The present results come from the discourses of FPs who agreed to participate in the discussion groups. Although this presupposes towards attitudes of collaboration and availability that may not be generalized, it adds a special value to the study of their perceptions. Their different origins and degree of knowledge about EBM and their extensive work experience enabled an in-depth search of shared opinions and revealed new areas for debate.

### EBM and primary care: not only a question of barriers

Analysis of the participants' discourses gives a dynamic view toward implementing EBM in PC. As observed in previous studies, EBM is perceived as positive in most groups [8,9,11,14] Its interpretation, however, is not based on a precise definition of EBM but is constructed over time in daily practice from a set of experiences and issues concerning its usefulness.

Workload pressure, lack of time, limited computer expertise and training in EBM, and a lack of knowledge of English in our environment have also been identified in other studies as the main barriers to the implementation of EBM [8,11,14]. In our opinion these obstacles are closely related to access and management of information but cannot fully account for the limited incorporation of EBM in daily practice.

Implementation is also conditioned by what we denominated a "defensive reaction" whose origins were found mainly "outside" the consultation. The role of EBM as a "reactive" tool to the pressure of the pharmaceutical industry, to its connection with money saving and managerial policies, and to its use to neutralize the influence of other specialists, the media and even the claims of patients, together create a scenario that goes beyond the search for information [17,28]. It would therefore be reasonable to question whether the term "barriers" is the most appropriate summary-metaphor of the difficulties for change in the EBM field [29]. The "reactive" use of EBM can determine not only the answers but also the FPs' questions themselves, or in other words, the kind of demands for using the EBM can condition the kind of resultant EBM. Decisions about which type of intervention, medication or care should be reviewed and evaluated on the basis of EBM appear to be conditioned by the above factors, all of which contribute to "EBM sensemaking" in PC. Connecting the mentioned "reactive" focus with the context in which EBM is developed opens the debate towards health policies and organizational strategies [3]. In addition to measures intended to improve the search and management of information, a "pro-active" development of EBM would be advisable to respond to the specific priorities of PC. Good patient care should be the bottom line when it comes to deciding which questions need to be answered and determining the criteria for their assessment.

### Science, EBM, and the new experts

For FPs EBM represents a model of care that is based on "science" and it is seen as connected to objectivity, publication and numbers. However, in view of the limited recognition of empirically important aspects such as empathy, individualization, continuing patient-doctor relationship, and the lack of CPG with specific recommendations for the different kinds of patients, FPs consider EBM is only a part of what could be considered optimal clinical practice [12,30]. They consider "experience" is also an essential part and that it is not taken into account in EBM or "science". This exclusion generates discomfort and tensions in the FPs' discourses in a similar sense described by Cassell regarding clinicians' roles as scientists and care-givers [31]. Many of the contents related to "experience" can also concur with the features of "patient-centered medicine" considered by authors like Benzing [32], as a different, but complementary paradigm to EBM. This questioning reflects the predominance of a view of science and evidence which excludes, or relegates to the lowest level in the hierarchy, many typical PC actions that are based on values or that cannot be experimentally studied. The need to develop other views of evidence according to the complexity of clinical practice emerges as a challenge for PC [12,13,33,34].

Our findings also suggest that certain components of scientific practice, such as reflection and critical questioning, appear to be at risk. The demand for clear and practical "cookbook" type CPGs can be insufficient for the implementation of EBM [19,35] at least as a method and attitude for searching and appraising the best for patients' care. The delegation to "make EBM" to the "new evidence experts", more or less closer to FPs but on a different status, also leads us to wonder whether questioning the texts and experts' authority, as a foundation of EBM, would be in danger of being substituted in practice by new text formats and a new experts' authority.

Under such circumstances, and keeping in mind the marked influence of the pharmaceutical industry, management health policies, and other professional lobbies, the frequent mention of contradictory recommendations "based on the best evidence" inevitably conditions the "tendency to doubt" the objectivity and neutrality of the new experts [20], and indirectly, of EBM itself. Repercussions are important because of their possible paradoxical effect as an excuse for not changing.

Besides transparency in the production of GPCs and other EBM tools [20,36], we need to promote deeper debate among FPs on the theoretical basis of EBM [4,37,38] and its role in real clinical practice [39] in order to improve good patient care in PC.

## Conclusions

The meaning of EBM is dynamically constructed by FPs within the primary care context from the experiences concerning its usefulness in their daily practice. EBM, as a model of care based on "science", represents only a part of good clinical practice. "Experience" emerges also as another essential part that resumes many typical PC actions that are based on values or that cannot be experimentally studied. The current views of evidence and science among the FPs should be discussed and developed according to the complexity of PC.

Difficulties for change in the EBM field go beyond the "barriers" related to lack of time, training and information access. The use of EBM is also conditioned by its role as a "defensive tool" faced with the pressure of the pharmaceutical industry, with its connection with money saving and managerial policies, and with its use to neutralize the influence of other specialists, the media and even the claims of patients. This "reactive" EBM sensemaking can determine not only the answers but also the questions addressed to EBM. A more "pro-active" and patient-centered development of EBM would be advisable at PC level.

Along with concerns about its objectivity, there arises a tendency to see EBM as the use of simplified guidelines developed by EBM experts. Contradictory recommendations "based on the best evidence" condition a "tendency to doubt" about the objectivity and neutrality of EBM. Transparency in the production of GPCs and other EBM tools comes up as necessary, and also it emerges the question whether some of the theoretical foundations of EBM are in danger of being substituted by new text formats and a new profile of experts.

## List of Abbreviations used

(EBM): Evidence based medicine; (FPs): Family physicians; (CPG): Clinical practice guidelines; (PC): Primary care.

## Competing interests

Authors Pablo Alonso-Coello, Rafael Rotaeche and Mercè Marzo-Castillejo are members of the Evidence-Based Medicine Working Group of the Spanish Association of Family and Community Medicine (semFYC).

## Authors' contributions

CC, PAC, IS and RR conceived the study design and performed the analysis of focus groups. CC, PAC, IS, RR, RC and AIG helped to organise the various groups and collaborated in data collection. CC developed the first draft of the manuscript. CC and PAC were responsible for reading and checking the manuscript before the submission. All authors participated in the discussion of the analysis, the editing of the drafts and read and agreed on the final version of this manuscript.

## Pre-publication history

The pre-publication history for this paper can be accessed here:

http://www.biomedcentral.com/1471-2296/12/84/prepub
